# NMDA receptor subunits have different roles in NMDA-induced neurotoxicity in the retina

**DOI:** 10.1186/1756-6606-6-34

**Published:** 2013-07-31

**Authors:** Ning Bai, Tomomi Aida, Michiko Yanagisawa, Sayaka Katou, Kenji Sakimura, Masayoshi Mishina, Kohichi Tanaka

**Affiliations:** 1 Laboratory of Molecular Neuroscience, Medical Research Institute, Tokyo Medical and Dental University, 1-5-45 Yushima, Bunkyo-ku, Tokyo 113-8510, Japan; 2 The Center for Brain Integration Research, Tokyo Medical and Dental University, Tokyo, Japan; 3 JST, CREST, Saitama, Japan; 4 College of Basic Medicine, China Medical University, 92 Bei Er Road, Heping District, Shenyang 110001, China; 5 Department of Cellular Neurobiology, Brain Research Institute, Niigata University, Niigata 951-8585, Japan; 6 Brain Science Laboratory, The Research Organization of Science and Technology, Ritsumeikan University, Nojihigashi 1-1-1, Kusatsu, Shiga 525-8577, Japan

**Keywords:** NMDA receptor, GluN2B, GluN2D, Excitotoxicity, Retina, Glaucoma, Glutamate transporter

## Abstract

**Background:**

Loss of retinal ganglion cells (RGCs) is a hallmark of various retinal diseases including glaucoma, retinal ischemia, and diabetic retinopathy. N-methyl-D-aspartate (NMDA)-type glutamate receptor (NMDAR)-mediated excitotoxicity is thought to be an important contributor to RGC death in these diseases. Native NMDARs are heterotetramers that consist of GluN1 and GluN2 subunits, and GluN2 subunits (GluN2A–D) are major determinants of the pharmacological and biophysical properties of NMDARs. All NMDAR subunits are expressed in RGCs in the retina. However, the relative contribution of the different GluN2 subunits to RGC death by excitotoxicity remains unclear.

**Results:**

GluN2B- and GluN2D-deficiency protected RGCs from NMDA-induced excitotoxic retinal cell death. Pharmacological inhibition of the GluN2B subunit attenuated RGC loss in glutamate aspartate transporter deficient mice.

**Conclusions:**

Our data suggest that GluN2B- and GluN2D-containing NMDARs play a critical role in NMDA-induced excitotoxic retinal cell death and RGC degeneration in glutamate aspartate transporter deficient mice. Inhibition of GluN2B and GluN2D activity is a potential therapeutic strategy for the treatment of several retinal diseases.

## Background

Glutamate is the major excitatory neurotransmitter in the mammalian central nervous system. However, its accumulation in extracellular spaces kills neurons through excitotoxic mechanisms via activation of glutamate receptors [1]. Excitotoxic neuronal cell death is thought to be a final common pathway in various neurological diseases, ranging from acute ischemic stroke to chronic neurodegenerative diseases such as Alzheimer’s disease and amyotrophic lateral sclerosis [2-5]. Glutamate excitotoxicity has also been proposed to be an important contributor to the death of retinal ganglion cells (RGCs) in glaucoma and ischemia-related conditions such as vessel occlusion and diabetic retinopathy [6-8], although some investigations have failed to confirm elevated glutamate concentration both in human patients with glaucoma [9] and in animal models of glaucoma [10,11]. The toxic effects of glutamate on RGCs are predominantly mediated by the overstimulation of N-methyl-D-aspartate (NMDA)-type glutamate receptors (NMDARs) due to their extreme permeability to calcium ions [12].

NMDARs are composed of various combinations of GluN1 and GluN2 (GluN2A–GluN2D) subunits and, in some cases, GluN3 (GluN3A and GluN3B) subunits. GluN2 subunits are major determinants of the functional properties of NMDARs, including characteristics such as agonist affinity, deactivation kinetics, single-channel conductance, Ca^2+^ permeability, and sensitivity to Mg^2+^[13]. However, the relative contribution of different GluN2 subunits to RGC death by excitotoxicity remains unclear.

We previously reported that NMDAR-mediated excitotoxicity contributed to the degeneration of RGCs in glutamate aspartate transporter (GLAST) deficient (KO) mice, the first animal model of normal tension glaucoma [14]. Furthermore, we recently reported that GluN2D deficiency partially protected against the loss of RGCs in GLAST KO mice [15]. These results suggest that other GluN2 subunits, in addition to GluN2D, may contribute to excitotoxic retinal cell death. To address this hypothesis, we examined the roles of the four different GluN2 subtypes in NMDA-induced retinal cell death using mice lacking specific GluN2 subunits. We also evaluated the neuroprotective effect of 7-hydroxy-6-methoxy-2-methyl-1-(2-(4-(trifluoromethyl)phenyl)ethyl)-1,2,3,4-tetrahydroisoquinoline hydrochloride (HON0001) [16], an specific GluN2B antagonist, on RGC degeneration due to glutamate excitotoxicity in GLAST KO mice.

In the present study, we report that GluN2B and GluN2D deficiency protect against NMDA-induced excitotoxic retinal cell death, but GluN2A and GluN2C deficiency have no protective effects. We also show that pharmacological blockade of GluN2B subunit attenuates RGC loss in GLAST KO mice.

## Results

### NMDA receptor subunits present in mouse RGCs

**Figure 1:**
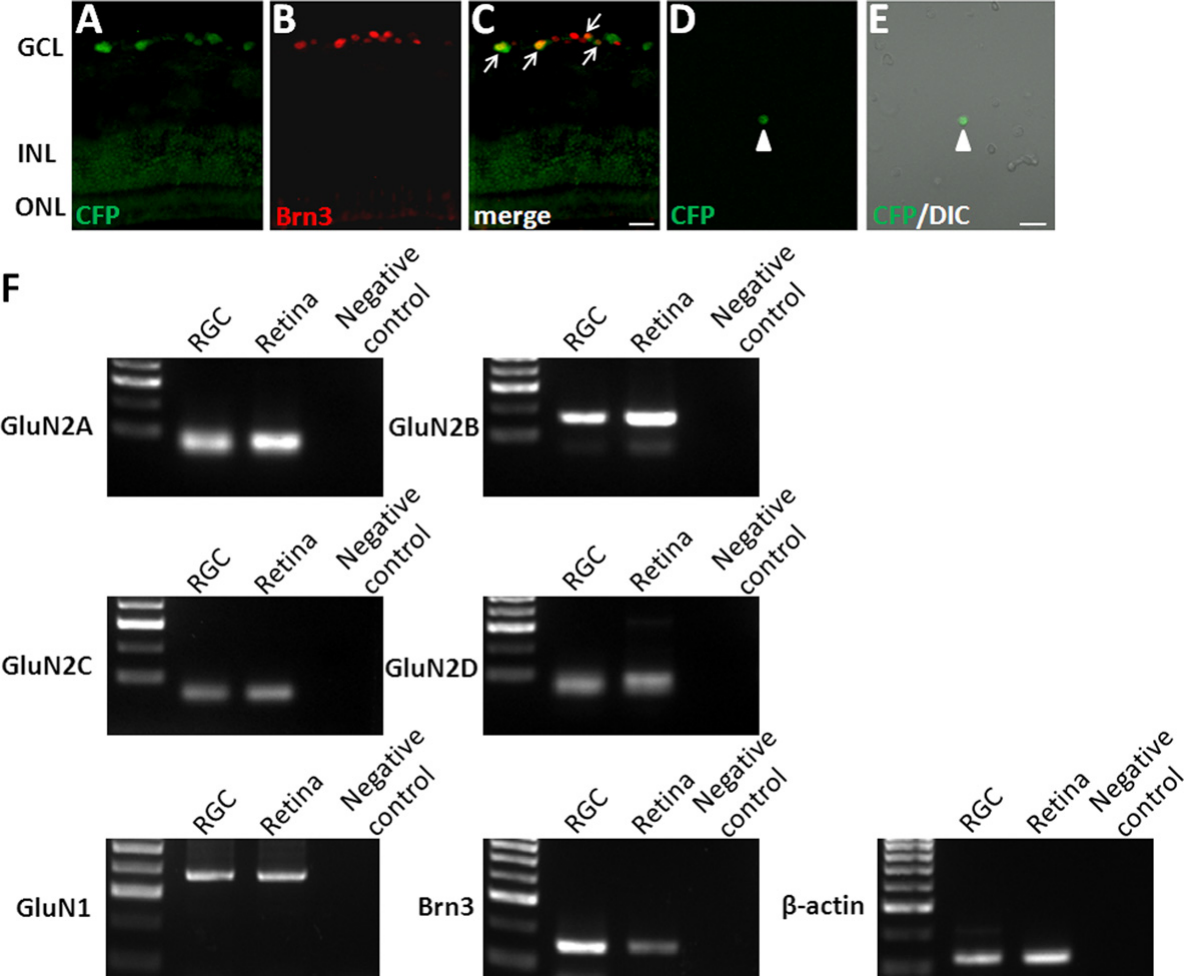
**Expression of NMDA receptor subunits in mouse retinal ganglion cell. (A-C)** Immunohistochemical analysis of Brn3 (**B** red) in Thy1-CFP mice. CFP fluorescence (**A** green) was overlaid with Brn3 **(C)**. Arrows in **(C)** indicate double-labeled cells. Scale bar, 20 μm. **(D-E)** After dissociation the fluorescent RGC was picked up from the cell suspension. CFP (green) and DIC pictures for the same isolated cell are superimposed **(E)**. Arrowhead indicates CFP-expressing RGC. Scale bar, 20 μm. **(F)** Single-cell RT-PCR analysis for GluN1, four GluN2 subunits, Brn3 and β-actin. Distilled water was used for PCR negative control. GCL, ganglion cell layer; INL, inner nuclear layer; ONL, outer nuclear layer; RGC, retinal ganglion cell.

To investigate the expression of NMDAR subunits in RGCs, we used a single-cell reverse transcriptase polymerase chain reaction (RT-PCR) method. After dissociation of the retina into single cells, RGCs can no longer be identified by their morphology. We therefore used dissociated retina from B6.Cg-TgN(Thy1-CFP)23Jrs/J transgenic mice (thy1-CFP mice), which express cyan fluorescent protein (CFP) in most RGCs [17]. We first confirmed that the CFP-containing cells in the thy1-CFP mouse retina were RGCs by immunostaining with Brn3, a neurochemical marker for RGCs [18]. CFP expression colocalized with Brn3 immunoreactivity in most somata in the ganglion cell layer (GCL) (Figure 1A-C). A single CFP-expressing cell was picked with a glass capillary from the dissociation mix and transferred to the reaction tube (Figure 1D, E), and was further identified as RGC by expression of Brn3 (Figure 1F). Typical results of single-cell RT-PCR on isolated RGCs are shown in Figure 1F. GluN1 and GluN2A–D could be amplified together with an internal control (β-actin) from a single RGC, as well as from whole retina. In our samples of 4 isolated RGCs, two cells express GluN1/GluN2A/GluN2B/GluN2C/GluN2D, whereas the other two cells express GluN1/GluN2A/GluN2B/GluN2D. These results indicate the presence of GluN1 and all GluN2 subunits (GluN2A–D) in the mouse RGCs.

### Retinal structure in mice lacking GluN2 subunits

**Figure 2:**
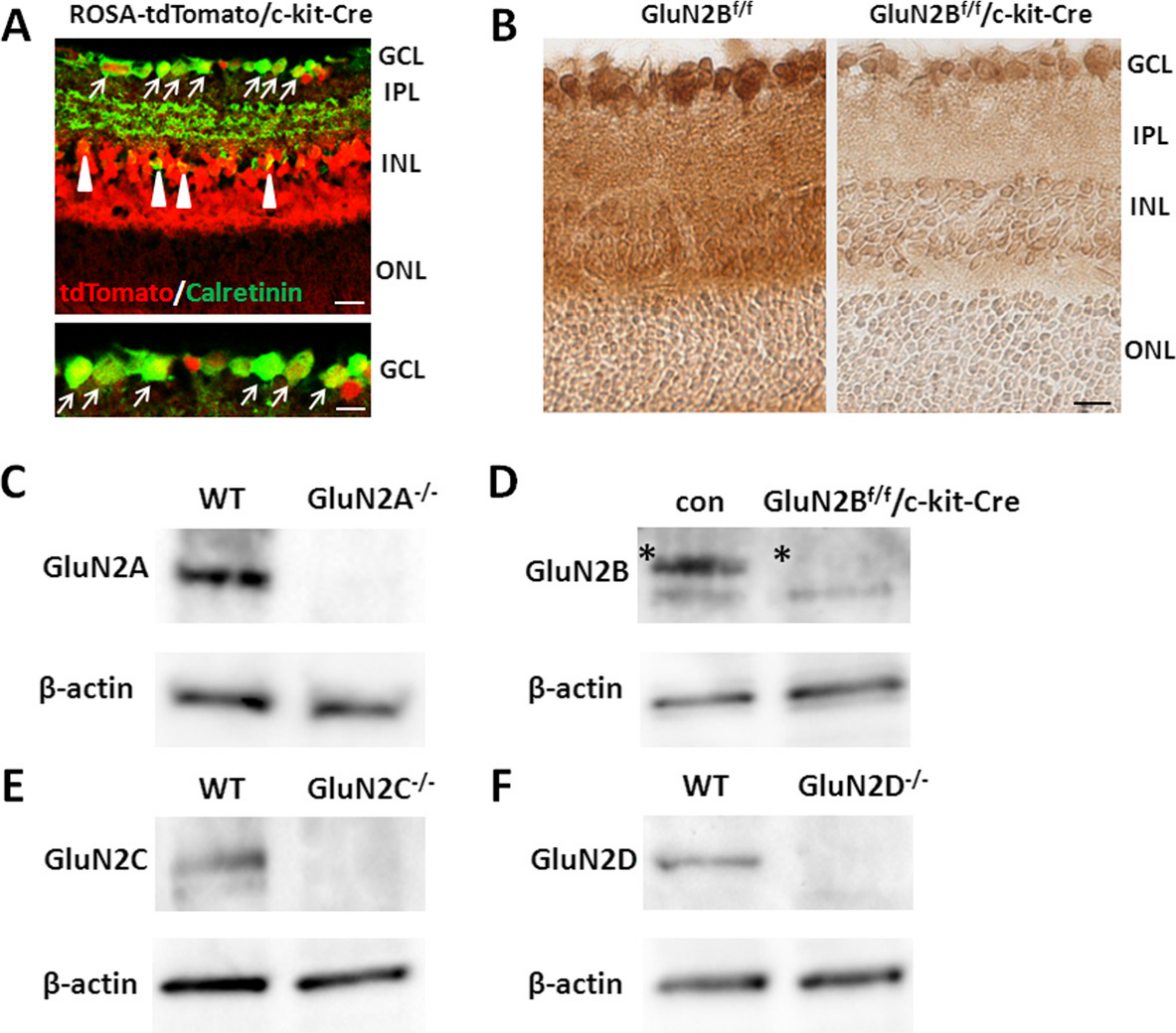
**Ablation of GluN2 subunits in the retinas of mutant mice. (A)** Immunostaining of calretinin (green) in ROSA-tdTomato/c-kit-Cre mice. Overlapping of tdTomato fluorescence (red) and calretinin indicated that Cre-mediated recombination occurs in RGCs (arrows) and amacrine cells (arrowheads). Enlarged image of the GCL in the upper panel was shown. Scale bar, 20 μm (upper) and 10 μm (lower). **(B)** Immunohistochemical analysis of GluN2B in GluN2B^f/f^ and GluN2B conditional knockout mice (GluN2B^f/f^/c-kit-Cre). Scale bar, 20 μm. **(C-F)** Western blot analysis of retinas from WT and GluN2 mutant mice with respective antibodies (GluN2A, GluN2B, GluN2C, GluN2D and β-actin). For GluN2B, control (con) represents GluN2B^f/f^ mice. Asterisks indicate the GluN2B protein bands. Each lane was loaded with 30 μg of proteins. GCL, ganglion cell layer; IPL, inner plexiform layer; INL, inner nuclear layer; ONL, outer nuclear layer.

We used mice lacking any one of the four GluN2 subunits to determine the distinct roles of these GluN2 subunits in NMDA-induced RGC death. Mice lacking GluN2A, GluN2C, and GluN2D are viable [19-21], whereas GluN2B-deficient mice die shortly after birth [22]. We therefore generated conditional GluN2B KO mice, in which GluN2B was ablated in retinal neurons containing RGCs. For this purpose, we crossed GluN2B^f/f^[23] mice with c-kit-Cre mice [24] (GluN2B^f/f^/c-kit-Cre). In c-kit-Cre mice crossed with ROSA-tdTomato reporter mice [25] (ROSA-tdTomato/c-kit-Cre), tdTomato-expressing cells were localized in the GCL and inner nuclear layer (INL) and most calretinin immunoreactive cells (RGCs and amacrine cells) contained tdTomato, suggesting that Cre recombinase is expressed in RGCs and cells in the INL, including amacrine cells, in c-kit-Cre mice (Figure 2A). Immunohistochemical analysis revealed that GluN2B protein expression was eliminated in RGCs and cells in the INL in GluN2B^f/f^/c-kit-Cre mice (Figure 2B). Western blot analysis showed that GluN2A, GluN2C, and GluN2D proteins were completely eliminated from mutant mice lacking GluN2A, GluN2C, and GluN2D, respectively (Figure 2C, E, F). In GluN2B^f/f^/c-kit-Cre mice, GluN2B expression level in the retina was significantly lower than in control mice (Figure 2D).

**Figure 3:**
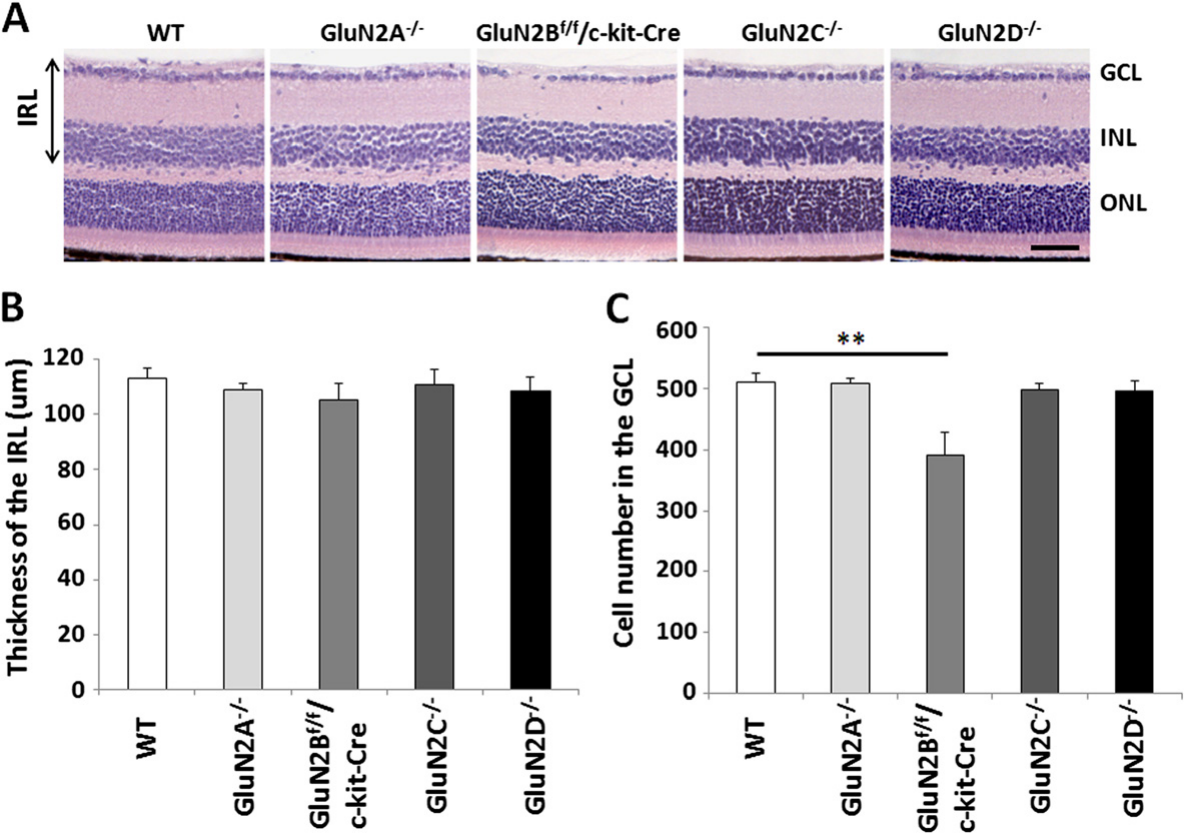
**Effects of GluN2 subunits ablation on the morphology of the retina. (A)** Hematoxylin and eosin staining (H&E) of retinal sections at P35 in WT and GluN2 mutant mice. Scale bar, 50 μm. **(B-C)** Quantification of thickness of the inner retinal layer **(B)** and the cell number in the GCL **(C)** in WT and GluN2 mutant mice. The data are presented as mean ± S.E.M. of 5 samples for each experiment. **P <0.01. GCL, ganglion cell layer; INL, inner nuclear layer; ONL, outer nuclear layer; IRL, inner retinal layer.

We next investigated whether the absence of GluN2 subunits affects the anatomical organization of the retina by histological analyses. Hematoxylin and eosin staining revealed the retinae of GluN2A, GluN2B^f/f^/c-kit-Cre, GluN2C, and GluN2D mutant mice to be normally organized, consisting of several different cell layers (Figure 3A). The thickness of the inner retinal layer (IRL) in all mutant strains was normal compared with wild-type (WT) mice (Figure 3B). As previous studies showed that ablation of GluN1 increased cell death in the developing somatosensory thalamus [26], we counted cell numbers in the GCL. The cell number in the GCL of GluN2B^f/f^/c-kit-Cre mice was significantly lower than that of WT mice at 5 weeks, whereas cell number in the GCL of the other mutant strains was comparable to that of control mice at 5 weeks (Figure 3C). These results suggest that GluN2B subunit plays a survival role for RGCs during retinal development, but the other GluN2 subunits (GluN2A, GluN2C and GluN2D) are not involved in retinal development and survival in RGCs.

### GluN2B and GluN2D deficiency prevents NMDA-induced-excitotoxic retinal cell death

**Figure 4:**
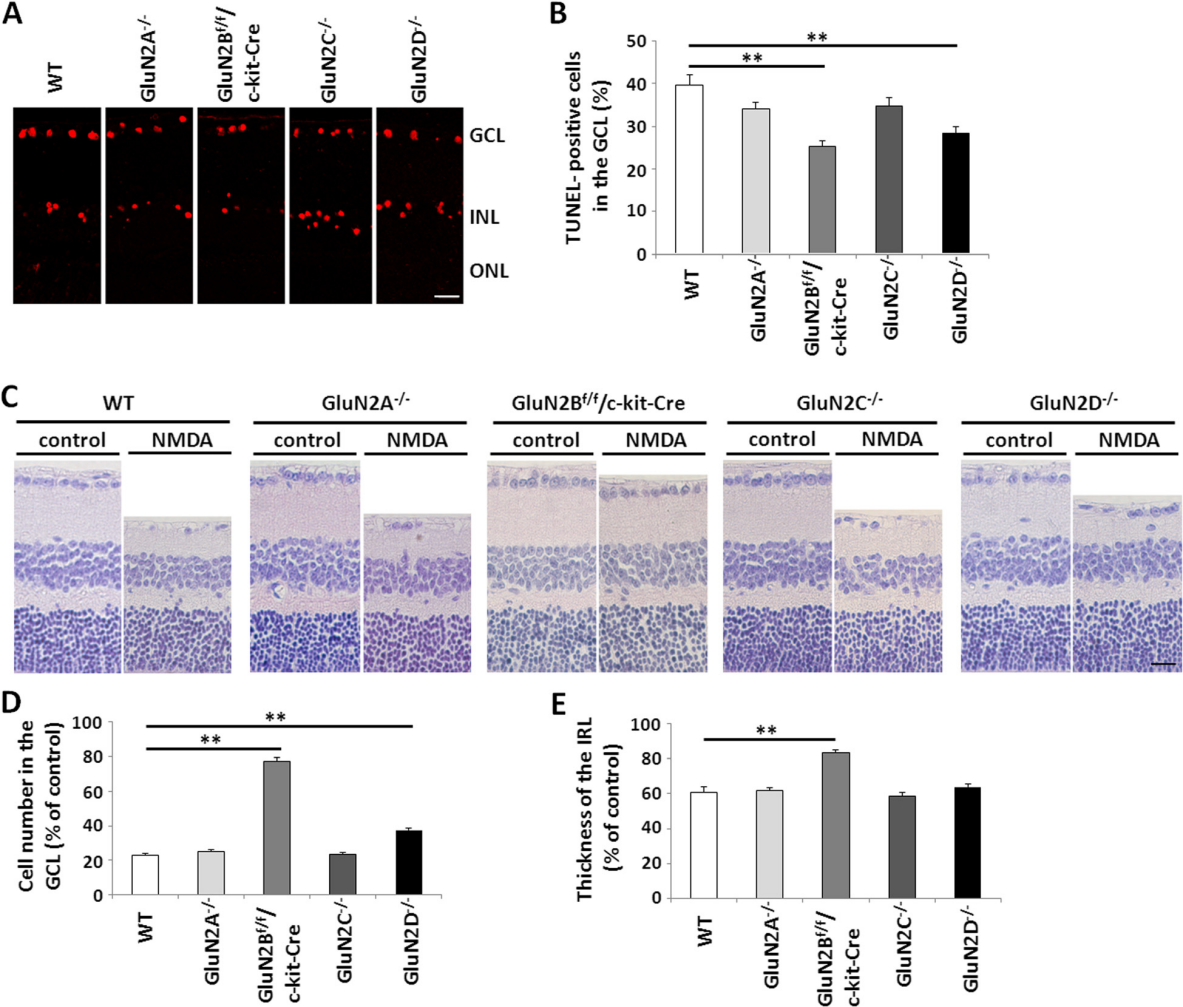
**TUNEL staining and morphometric analysis following NMDA treatment. (A)** Representative photos of TUNEL staining at 1 day after NMDA treatment from WT and GluN2 mutant mice. Scale bar, 20 μm. **(B)** Quantification of TUNEL-positive cells in the GCL. The data are presented as mean ± S.E.M. of 5 samples for each experiment. **P <0.01 **(C)** Representative photos of HE staining at 7 days after NMDA and PBS treatment from WT and GluN2 mutant mice. Scale bar, 20 μm. **(D-E)** Quantification of cell number in the GCL **(D)** and thickness of IRL **(E)**. The data are presented as mean ± S.E.M. of 5 samples for each experiment. **P <0.01. GCL, ganglion cell layer; INL, inner nuclear layer; ONL, outer nuclear layer; IRL, inner retinal layer.

To determine which GluN2 subtypes are involved in NMDA-induced RGC death in the retina, we examined the effect of intraocular injection of NMDA on retinal cell death in GluN2 KO and WT mice. First, to examine the acute injury of NMDA, TUNEL analysis was performed on the retinas of WT and GluN2 mutants at 1 day after NMDA treatment. A number of TUNEL-positive cells were observed in the GCL and INL in both WT and GluN2 mutant strains after NMDA injection (Figure 4A), but the percentage of TUNEL-positive cells in the GCL of GluN2B^f/f^/c-kit-Cre and GluN2D^−/−^ mice was significantly lower than that in WT mice (Figure 4B). Following NMDA injection, the number of RGCs and the thickness of IRL decreased from days 1 to 7, with no further decrease being observed from days 7 to 14 [27,28]. To examine the chronic injury of NMDA, morphological changes were measured 7 days after NMDA or phosphate-buffered saline (PBS) injection. Intraocular administration of NMDA induced cell death in the GCL in both WT and GluN2 mutant mice (Figure 4C), but the percentage of surviving cells in the GCL was significantly larger in GluN2B^f/f^/c-kit-Cre and GluN2D^−/−^ mice than in WT mice (Figure 4D). Additionally, the thickness of IRL was significantly larger in GluN2B^f/f^/c-kit-Cre mice than in WT mice (Figure 4E). Taken together, these results suggest that GluN2B and GluN2D were involved in NMDA-induced RGC death.

### A specific GluN2B antagonist, HON0001, prevents RGC death in GLAST-deficient mice

**Figure 5:**
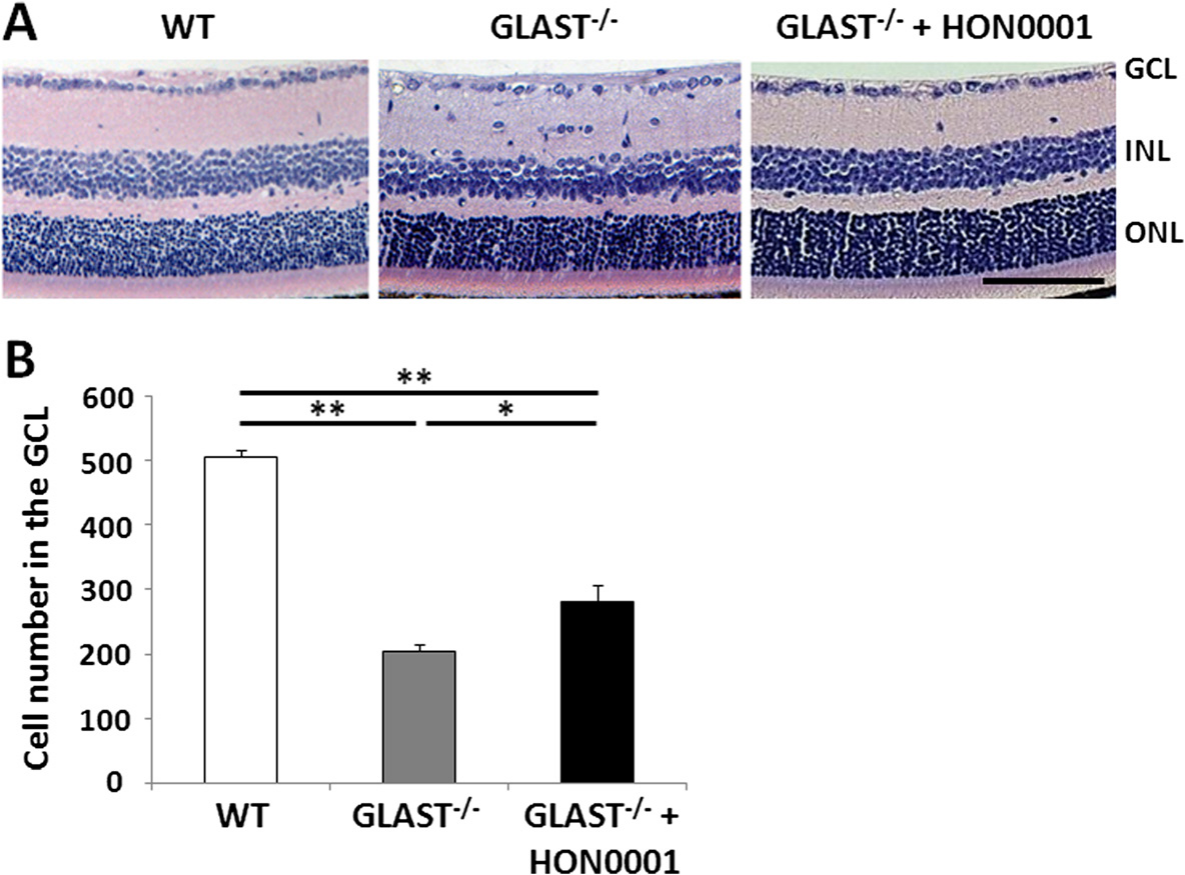
**GluN2B antagonist HON0001 rescues RGCs death in GLAST-deficient mice. (A)** Representative photos of wild-type (WT), saline- (GLAST^−/−^) or HON0001- (GLAST^−/−^ + HON0001) treated retinas. HON0001 (10 mg/kg) or saline were injected orally (p.o.) into GLAST^−/−^ mice from P21 to P35. The animals were killed at P35 after HON0001/saline treatment. Scale bar, 100 μm. **(B)** Quantification of the cell number in the GCL. The data are presented as mean ± S.E.M. of 4 (WT and GLAST^−/−^) and 6 (GLAST^−/−^ + HON0001) samples for each experiment. *P <0.05, **P <0.01. GCL, ganglion cell layer; INL, inner nuclear layer; ONL, outer nuclear layer.

We have reported that the neuroprotective role of apolipoprotein E-containing lipoproteins in glaucomatous retinal degeneration in GLAST KO mice is mediated through promoting interaction between low density lipoprotein receptor-related protein 1 (LRP-1) and the GluN2B subunit [29]. Recently, we have also demonstrated that Dock3 overexpression prevented retinal cell death in GLAST KO mice by promoting GluN2B degradation [28]. To determine whether GluN2B is involved in RGC degeneration in GLAST-deficient mice, we evaluated the effect of a specific GluN2B antagonist, HON0001, on RGC degeneration in GLAST KO mice. As shown in Figure 5, the number of cells in GLAST KO mice subjected to HON0001 (10 mg/kg) treatment (281 ± 26) was significantly greater than that in GLAST KO mice not subjected to HON0001 treatment (203 ± 10). These results suggest that GluN2B is involved in RGC loss in GLAST KO mice.

## Discussion

We previously reported that GluN2D deficiency prevented loss of RGCs in GLAST KO mice [15]. These results suggest that both GluN2B and GluN2D subunits play a critical role in RGC degeneration by glutamate excitotoxicity. Therefore, an GluN2B-selective antagonist in combination with an GluN2D-selective antagonist represents an effective strategy for the management of glaucoma and various forms of retinopathy. We recently showed that Dock3 overexpression prevented excitotoxic RGC death by suppressing the surface expression of GluN2D and enhancing NMDA-mediated GluN2B degradation [15,28]. Thus, the design of compounds capable of increasing the expression of Dock3 represents a novel strategy for the treatment of various forms of retinopathy. Previous studies also showed that calcium influx through NMDARs is modulated by LRP-1 [30,31]. These findings may provide a novel therapeutic strategy for various forms of retinopathy that are mediated by E-containing lipoproteins through LRP-1.

The failure of GluN2C deficiency to protect RGCs from NMDA-induced excitotoxicity can be explained by the data showing that only a small number of RGCs expressed GluN2C [32]. However, almost RGCs express GluN2A [32]. The failure of GluN2A deficiency to protect RGCs from NMDA-induced excitotoxicity may be explained by the distinct functional properties conferred by GluN2 subunits on the receptors, and the different signaling pathway couplings [13,33]. This variety is due to the large and divergent cytoplasmic C-terminal domains of GluN2 subunits [34]. A previous report showed that C-terminal domains of GluN2B subunits were more lethal than GluN2A subunits, and different coupling to PSD-95/nNOS signaling cassette may contribute to differential susceptibility of GluN2 subunits to excitotoxic injury [33]. Another possible explanation is that the location of NMDARs at synaptic or extrasynaptic sites determines the neuroprotective or neurotoxic effects of glutamate. A high level of synaptic NMDAR activity promotes neuronal survival, whereas extrasynaptic NMDAR activity promotes cell death [35]. In the retina, GluN2B is enriched at the perisynaptic site, whereas synaptic NMDARs primarily contain GluN2A [36].

The number of cells in the GCL of GluN2B^f/f^/c-kit-Cre mice was significantly decreased at 5 weeks. This finding is consistent with a previous study showing that NMDAR hypofunction increased neuronal death in the developing brain [26,37]. GluN2B is a major GluN2 subunit in the immature retina [38]; therefore, ablation of GluN2B in the developing retina can cause excessive neuronal apoptosis, resulting in a reduction in the cell number in the GCL of GluN2B^f/f^/c-kit-Cre mice. Thus, loss of GluN2B can increase RGC death in the immature retina, but protect RGCs from glutamate excitotoxicity in the adult.

## Conclusions

We showed that GluN2B- and GluN2D-containing NMDARs played a critical role in NMDA-induced excitotoxic retinal cell death and RGC degeneration in GLAST KO mice. Inhibition of GluN2B and GluN2D activity is a potential therapeutic strategy for the treatment of several retinal diseases, including retinal ischemia, diabetic retinopathy, and glaucoma.

## Methods

### Animals

B6.Cg-TgN(Thy1-CFP)23Jrs/J transgenic mice (thy1-CFP mice) and c-kit-Cre transgenic mice have been described previously [17,24]. c-kit-Cre transgenic mice were bred with ROSA-tdTomato mice [25] to examine Cre activity. c-kit-Cre mice were bred with GluN2B^flox/flox^ (*GluN2B*^*f/f*^) mice [23] to generate GluN2B conditional knockout mice (*GluN2B*^*f/f*^*/c-kit-Cre*). The homozygous GluN2A KO (*GluN2A*^*−/−*^) [19], GluN2C KO mice (*GluN2C*^*−/−*^) [20] and GluN2D KO mice (*GluN2D*^*−/−*^) [21] were obtained by crossing heterozygous GluN2A^+/−^, GluN2C^+/−^ and GluN2D^+/−^ mice, respectively. GLAST KO mice have been described previously [39,40]. In all experiments, age matched WT and GluN2B^f/f^ littermate controls were used. All mice were of the C57BL/6 J genetic background, and all animal procedures were approved by the Animal Committee of Tokyo Medical and Dental University (0130166C).

### Isolation of single ganglion cells from mouse retina and RT-PCR

5 week old Thy1-CFP mice were deeply anesthetized by diethyl ether and retinas were dissociated by using the Papain Dissociation System (Worthington Biochemical Corporation) at 37°C for 30 min. Single-CFP-expressing cell was aspirated by glass microcapillaries and placed into the PCR-tube containing 10 μl of resuspention buffer. Single-cell RT-PCR was performed using the SuperScript III CellsDirect cDNA Synthesis System (Invitrogen). Total RNA (5 μg) from whole retina were used to synthesize first-strand cDNA by using SuperScript III First-Strand Synthesis System (Invitrogen). The retina cDNA served as positive controls. The following primers were used for cDNA detection: GluN2A FWD: 5′ GTG TGC GAC CTC ATG TCC G 3′; REV: 5′ GCC TCT TGG TCC GTA TCA TCT C 3′; GluN2B FWD: 5′ CAG CAA AGC TCG TTC CCA AAA 3′; REV: 5′ GTC AGT CTC GTT CAT GGC TAC 3′; GluN2C FWD: ATC CCC GAC GGC TGA GA 3′; REV: 5′ TTC CTA GTC CAA GCA CAA AAC G 3′; GluN2D FWD: 5′ TGT GTG GGT GAT GAT GTT CGT 3′; REV: 5′ CCA CAG GAC TGA GGT ACT CAA AGA 3′; GluN1 FWD: 5′ GCC GAT TTA AGG TGA ACA GC 3′; REV: 5′ AAT TGT GCT TCT CCA TGT GC 3′; Brn3 FWD: 5′ GCA GTC TCC ACT TGG TGC TTA CTC 3′; REV: 5′ TTC CCC CTA CAA ACA AAC CTC C 3′; β-actin FWD: 5′ ATA TCG CTG CGC TGG TCG TC 3′; REV: 5′ TCA CTT ACC TGG TGC CTA GGG 3′. The thermal cycler conditions were 5 min at 94°C and then 40 cycles of 30 s at 94°C, 30 s at 60°C, and 30 s at 72°C, followed by 7 min at 72°C.

### Western blot analysis

Retinas were quickly removed and homogenized in 100 μl of cold lysis buffer (50 mM Tris–HCl, 1% Nonidet P-40, 5 mM EDTA, 150 mM NaCl, 0.5% Na-deoxycholate, 1 mM MgCl_2_, 1 mM DTT, 1 mM Na_3_VO_4_, 1 mM NaF, 1 mM phenylmethylsulfonyl fluoride (PMSF), and Complete Protease Inhibitor Cocktail [Roche]). Protein concentration was determined by BCA Protein Assay kit (Sigma-Aldrich). Thirty microgram of the protein was loaded per lane. Primary antibodies used were GluN2A (1:500, Covance), GluN2B (1:500) [41], GluN2C (1:100, Invitrogen), GluN2D (1:500) [42], β-actin (1:1000, Santa Cruz). They were then incubated with anti-rabbit, guinea pig or mouse IgG-HRP-conjugated secondary antibody (1:5000, Jackson ImmunoResearch). SuperSignal West Femto Maximum Sensitivity Substrate (Thermo Scientific) was used to visualize the immunoreactive proteins.

### Immunohistochemistry

Sections were prepared as previously described [15]. Frozen retinal sections of 12 μm thickness were incubated using anti-Brn3 (1:50, Santa Cruz), anti-calretinin (1:500, Swant) and anti-GluN2B (1:100) antibodies. For Brn3 and calretinin detection, Cy-3-conjugated donkey anti-goat IgG (1:500, Jackson ImmunoResearch) and goat anti-rabbit IgG Alexa 488 (1:1000, Molecular Probes) were used as secondary antibodies. For GluN2B detection, peroxidase labelled polymer conjugated to goat anti-rabbit IgG (DAKO) was used as secondary antibody. Images were recorded with an LSM-510 META confocal laser microscope (Carl Zeiss).

### Histology and morphometric analysis

Eyes from mice at postnatal day 35 (P35) were enucleated and fixed in Davidson’s solution fixative [43], then embedded in paraffin wax. In some experiments, HON0001 (10 mg/kg, a gift from T. Honda at Hoshi University) [16] or saline was injected orally (p.o.) into GLAST KO mice daily from P21 to P35. These mice were sacrificed on P35 and processed for RGC count. Paraffin sections (7 μm thick) were cut though the optic nerve and stained with hematoxylin and eosin (H&E). The number of neurons in the GCL was counted as previously described [15]. The thickness of the IRL (from GCL to INL) was measured at a distance of 0.5 to 1.0 mm from optic disc.

### Animal models of NMDA-induecd retinal neuronal death and morphometric analysis

Intravitreal injection of NMDA (Sigma) was conducted as previously described [15]. Briefly, a single 2-μl injection of 20 mM NMDA in 0.1 M PBS (pH 7.4) was administered intravitreally into the right eye of each mouse, the same volume of PBS was administered to the contralateral (left) eye as control. The animals were sacrificed at 1 day or 7 days after injection, and eyes were enucleated for morphometric and TUNEL analysis. Paraffin sections (5 μm thick) were cut though the optic nerve and stained with H&E. The extent of NMDA-induced retinal cell death after 7 days was quantified by counts of neurons in the GCL and the thickness of the IRL. The changes of the number of ganglion cells and thickness of IRL after NMDA injection were expressed as percentages of the control eyes.

### TUNEL staining

At 1 day after the NMDA or PBS injection, TUNEL staining was performed with paraffin sections (5 μm thick) according to the manufacturer’s instructions (Promega). Fluorescence detection was carried out using Alexa-fluor-568-conjugated streptavidin (Molecular Probes). TUNEL-positive cells in the GCL were counted and expressed as percentages of total DAPI stained cells in the GCL.

### Statistics

Statistical analyses were conducted using Student’s t-test for comparison between two samples, or one-way ANOVA followed by Bonferroni’s test for multiple comparisons, using the SPSS 17.0 software package. Data are expressed as mean ± S.E.M. *P* values < 0.05 were considered statistically significant.

## Abbreviations

GLAST: Glutamate aspartate transporter; INL: Inner nuclear layer; IPL: inner plexiform layer; IRL: Inner retina layer; LRP: lipoprotein receptor-related protein; NMDAR: N-methyl-D-aspartate receptor; PBS: Phosphate-buffered saline; PMSF: Phenylmethylsulfonyl fluoride; RGC: Retinal ganglion cell; RT-PCR: Reverse transcriptase polymerase chain reaction; WT: Wild-type.

## Competing interests

The authors declare that they have no competing interests.

## Authors’ contributions

KT, NB, and TA conceived and designed the experiments. NB, SK and MY carried out experiments and analyzed the data. KS and MM contributed reagents and materials. KT and NB wrote the paper. All authors have read and approved the manuscript for publication.
